# ESR1 Regulates Fecundity and Functions in Sheep Endometrial Stromal Cells

**DOI:** 10.3390/ijms262311410

**Published:** 2025-11-25

**Authors:** Kang Li, Xiaoxiao Gao, Zhibo Wang, Dongxu Li, Jiahe Guo, Feng Wang, Tianlong Guo

**Affiliations:** 1 Sanya Research Institute of Nanjing Agricultural University, Nanjing Agricultural University, Sanya 572000, China; 13947110359@163.com (K.L.); 2020205010@stu.njau.edu.cn (Z.W.); 2022205013@stu.njau.edu.cn (D.L.); gjh18753783920@163.com (J.G.); 2 Inner Mongolia Academy of Agricultural & Animal Husbandry Sciences, Hohhot 010030, China; 3 Jiangsu Livestock Embryo Engineering Laboratory, Nanjing Agricultural University, Nanjing 210095, China; 4 College of Animal Science and Technology, Qingdao Agricultural University, Qingdao 266109, China; gaoxiaoxiao2021@qau.edu.cn

**Keywords:** ESR1, proliferation, apoptosis, migration, endometrial stromal cells

## Abstract

Endometrial stromal cell (ESC) plasticity is critical to embryo survival and conceptus elongation; however, their genetic regulation remains unclear. To elucidate the molecular mechanisms by which ESR1 regulates sheep fecundity through endometrial function, we investigated the effects of ESR1 on ovine ESCs in vitro. Tissue distribution analysis revealed elevated ESR1 expression in the reproductive organs, particularly the endometrium, compared with that in non-reproductive tissues. Both ESR1 mRNA and its encoded ERα protein were significantly upregulated in the endometrium of high-fecundity Hu sheep compared to those in the endometrium of low-fecundity Hu sheep. Given the specific localisation of ERα in ESCs, we examined the functional roles of ESR1 in these cells in vitro. ESR1 knockdown in ESCs inhibited cell proliferation and induced apoptosis, concomitant with increased Caspase3 and Caspase9 mRNA expression. Furthermore, ESR1 interference triggered G0/G1-phase cell cycle arrest via the upregulation of P53 and P21 and significantly impaired cell migration capacity. Collectively, these results demonstrate that ESR1 critically regulates ESC proliferation, apoptosis, cell cycle progression, and migration. Our findings establish ESR1 as a key determinant of sheep fecundity and provide mechanistic insights into the high reproductive efficiency of Hu sheep.

## 1. Introduction

The reproductive efficiency of sheep is a primary determinant of flock profitability and global meat production. Among indigenous breeds, Hu sheep are renowned for their exceptionally large litter sizes (an average of 2.5–3.0 lambs per parturition); however, the molecular drivers of this prolificacy remain unclear. Endometrial receptivity and stromal cell (ESC) plasticity are rate-limiting factors in embryo survival and conceptus elongation; however, the genetic determinants governing these processes remain elusive.

The ESR1 gene encodes oestrogen receptor α (ERα), a subtype of oestrogen receptors (ERs) [1], predominantly expressed in the organs of the reproductive system, such as the hypothalamus, pituitary gland, uterus, cervix, vagina, and mammary gland [2]. ERα is a pivotal nuclear receptor that transduces oestradiol signals essential to uterine growth, stromal proliferation, and decidualization.

ESR1 is crucial in various physiological functions, such as reproduction. During reproduction, when ESR1 is knocked out in the uterine epithelium, no embryo implantation occurs following either natural mating or embryo transfer in mice, indicating its crucial role in establishing uterine receptivity [3]. Due to anovulation and a lack of corpora lutea, ESR1-null (αERKO) female mice are infertile and exhibit reproductive abnormalities by 50 days of age, such as increased gonadotropin and gonadotropin receptor levels and theca cell hypertrophy [4,5]. During the oestrus cycle of sheep, the effects of oestrogen and ERα are required for the development of the luteolytic mechanism in the epithelium of the uterine endometrial luminal and superficial ductal glands [6], and the expression level of ERα in the uterine artery endothelium during the luteal phase is 40% of that during the follicular phase [7,8]. In summary, ESR1 plays a key role in animal reproduction. Our earlier transcriptome analysis of the endometrial epithelium at the oestrus stage revealed the differential expression of ESR1 in high- and low-fertility Hu sheep. However, most studies have focused on ovarian or hypothalamic expression, and a direct functional link between endometrial ESR1 and fecundity has not yet been established.

ESCs are the principal cellular substrates for embryo attachment and subsequent placentation, and their proliferation, apoptosis, and migratory capacities are tightly regulated by steroid hormones. The dysregulation of ESC fate decisions has been implicated in early embryonic loss, recurrent implantation failure, and reduced litter size. Despite these clinical and economic implications, the molecular circuitry through which ESR1 modulates ESC function in sheep remains unclear.

To clarify the role of ESR1 in regulating ESC phenotypes, we conducted a multilevel investigation combining the transcript profiling of the Hu sheep endometrium with loss-of-function studies on primary ovine ESCs.

## 2. Results

### 2.1. Expression and Location of ESR1/ERα

**Figure 1:**
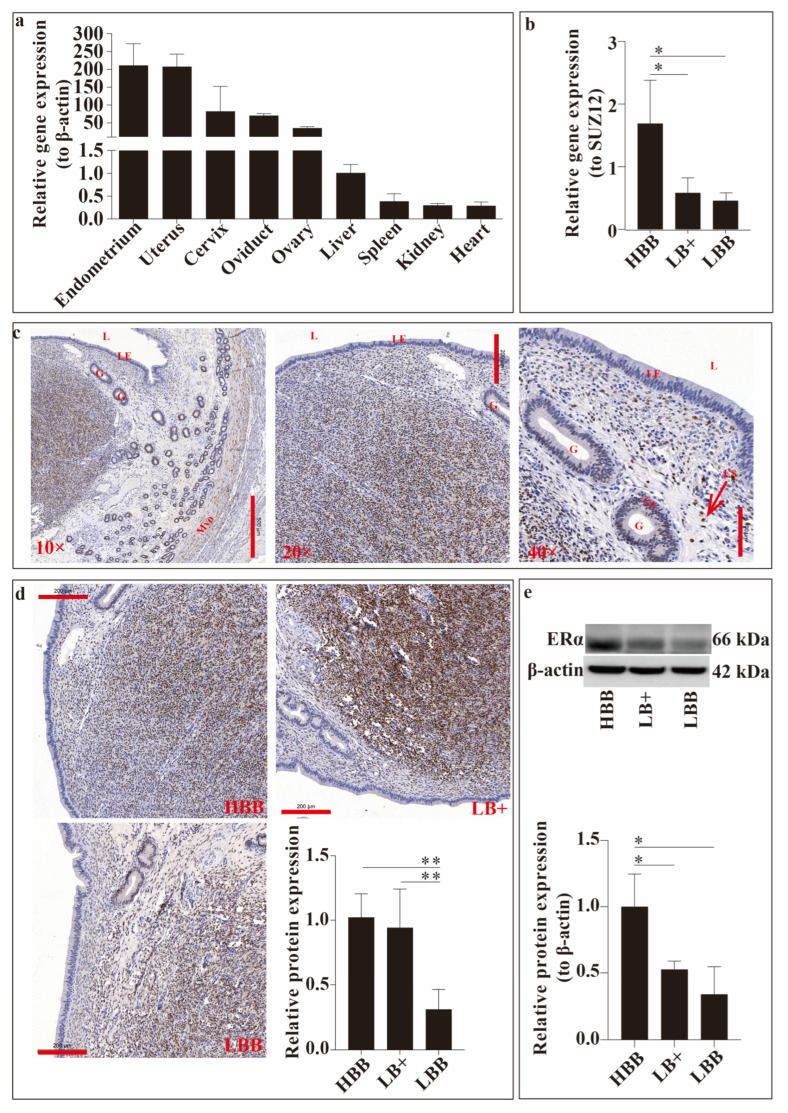
Expression analysis of ESR1/oestrogen receptor α (ERα) in Hu sheep during the oestrus period. (**a**) Relative expression of the ESR1 gene in the main visceral tissues. (**b**) Relative expression of the ESR1 gene in the endometrium with different fecundities. (**c**) Localisation of ERα in the uterine horn using immunohistochemical staining. Legend: L, lumen; G, gland; LE, luminal epithelium; ES, endometrial stroma; GE, glandular epithelium; Myo, myometrium. The scale bars of 10×, 20×, and 40× represent 500, 200, and 100 μm, respectively. (**d**) Relative expression of ERα protein in the uterus using immunocytochemical staining at the organisational level; the image is magnified 20×, and scale bars represent 200 μm. (**e**) Detection of the ERα protein in the endometrium with different fecundities at the molecular level. * *p* < 0.05; ** *p* < 0.01.

ESR1 expression was detected in the liver, heart, spleen, kidneys, cervix, uterus, oviduct, and ovary. As shown in Figure 1, the relative expression of ESR1 varied between samples. The relative expression level of ESR1 was observed to be highest in the uterus and endometrium and second highest in the cervix, oviduct, and ovary, followed by the liver, heart, spleen, and kidney (Figure 1a).

We compared the relative expression of ESR1 in Hu sheep endometria with different fecundities using real-time quantitative PCR (qRT-PCR) (Figure 1b). ESR1 expression in the HBB endometrium was significantly higher than that in the other groups (*p* < 0.05).

Subsequently, the localisation of ERα in the uterine horn was detected using immunocytochemistry staining (Figure 1c). ERα immunoreactivity was detected in the glandular epithelium, myometrium, and endometrial stroma of the uterus.

Finally, we compared endometrial ERα expression at the organisational and molecular levels in Hu sheep with different fecundities using immunocytochemical staining (Figure 1d) and Western blotting (Figure 1e). As shown in Figure 1d, ERα expression levels in the HBB and LB+ groups were significantly higher than those in the LBB group (*p* < 0.01). The same trend was observed at the molecular level (Figure 1e), and the expression level in the HBB group was significantly higher than that in the LB+ and LBB groups (*p* < 0.05).

### 2.2. Verification of ESR1 siRNA Transfection Efficiency

**Figure 2:**
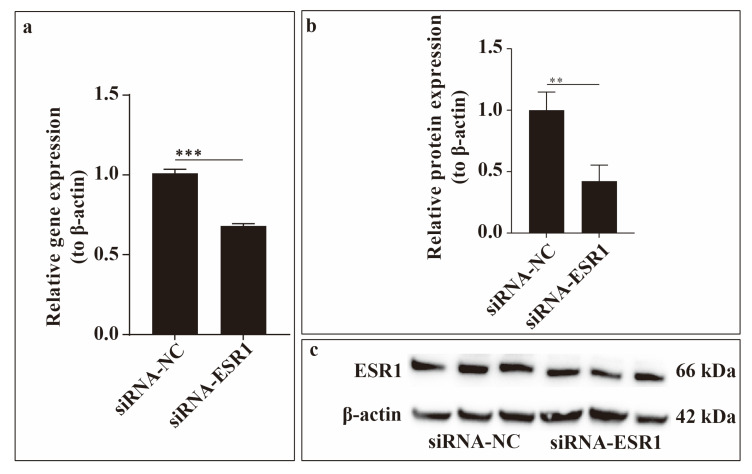
Transfection efficiency. Relative expression of ESR1 gene (**a**) and oestrogen receptor α (ERα) protein (**b**,**c**). ** *p* < 0.01; *** *p* < 0.001.

To investigate the effect of ESR1 inhibition on ESCs in vitro, we constructed siRNA-ESR1. We then performed ESR1 loss-of-function assays to validate transfection efficiency in ESCs. The results shown in the figure revealed that ESR1 expression was significantly decreased after siRNA-ESR1 transfection compared to the negative control (NC) at both the gene (detected using qRT-PCR) and protein (determined using Western blotting) levels (Figure 2). Therefore, siRNA-ESR1 was used in subsequent experiments.

### 2.3. ESR1 Knockdown Inhibits ESC Proliferation In Vitro

**Figure 3:**
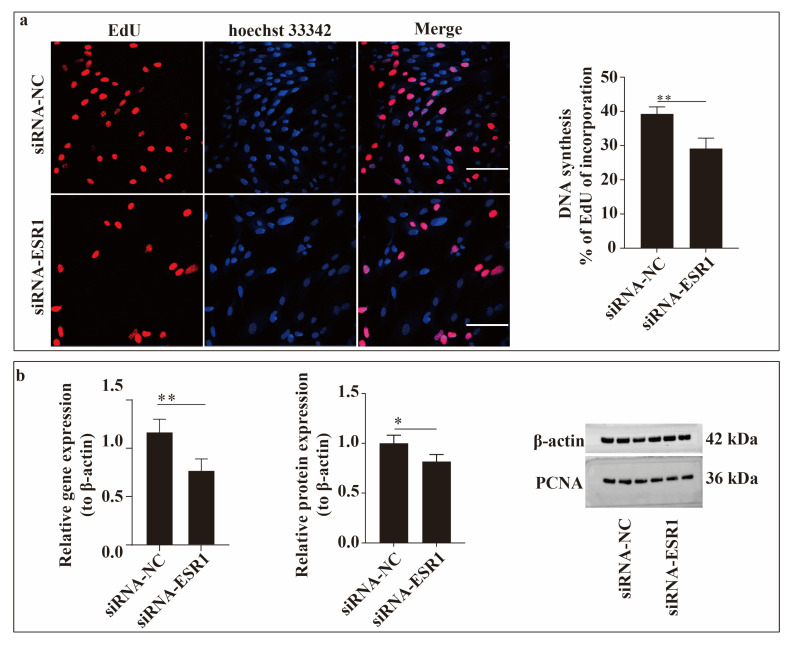
ESR1 function in endometrial stromal cell (ESC) proliferation in vitro. (**a**) Figure showing results of EdU experiment. Nuclear DNA was stained with Hoechst 33342 (blue), and proliferating cells were labelled with EdU (red). Scale bar = 100 μm. (**b**) Relative gene and protein expression levels of proliferating cell nuclear antigen (PCNA) in ESCs following siRNA-ESR1 transfection. * *p* < 0.05; ** *p* < 0.01.

To study the function of ESR1 in ESC proliferation in vitro, an EdU incorporation assay was conducted (Figure 3). As shown in Figure 3a, ESR1 knockdown significantly reduced (*p* < 0.01) the number of proliferating cells (*p* < 0.01). Additionally, the expression of proliferating cell nuclear antigen (PCNA), a key gene in cell proliferation, was detected at the gene (detected using qRT-PCR) and protein (determined using Western blotting) levels. The results for the two levels, shown in the figure, suggest that PCNA expression was downregulated (*p* < 0.05) when ESR1 expression was knocked down (Figure 3b). From these results, we can infer that downregulating the expression of ESR1 in ESC cultured in vitro inhibits cell proliferation and that ESR1 can promote cell proliferation.

### 2.4. ESR1 Knockdown Accelerated Cell Apoptosis and Arrested Cell Cycle Progress in Hu Sheep ESCs

**Figure 4:**
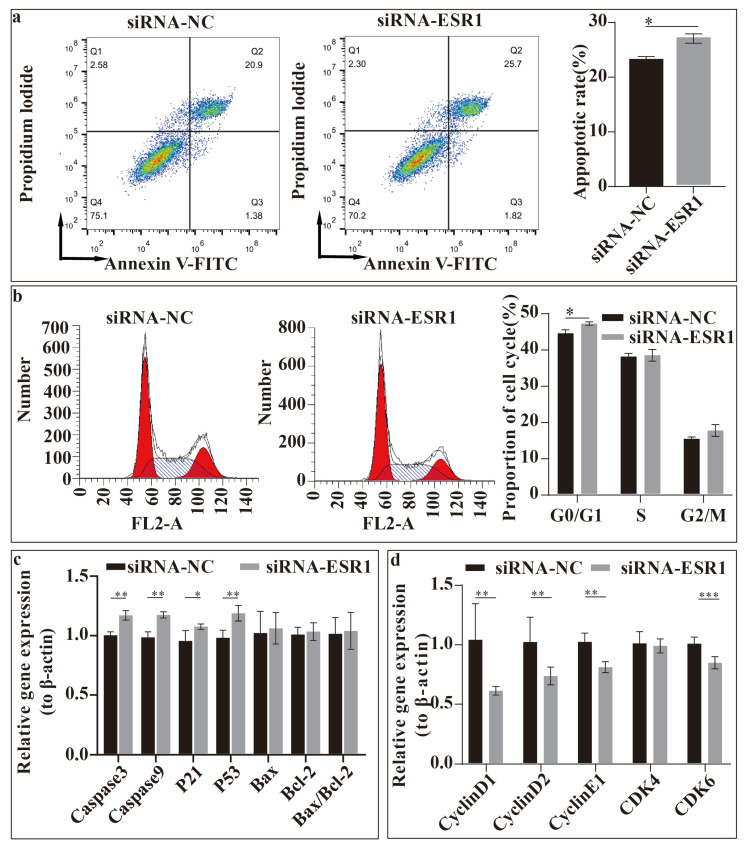
Effects of ESR1 gene on endometrial stromal cell (ESC) apoptosis and cell cycle progression in vitro. (**a**) Apoptosis rate in ESCs following ESR1 siRNA transfection. (**b**) Effects on cell cycle progression in ESCs following ESR1 siRNA transfection. (**c**) Relative expression levels of apoptosis-related genes in ESCs following ESR1 siRNA transfection. (**d**) Relative expression levels of cell cycle-related genes in ESCs following ESR1 siRNA transfection. * *p* < 0.05, ** *p* < 0.01, and *** *p* < 0.001.

Then, we studied the effect of the ESR1 gene on ESC apoptosis and cell cycle progress in vitro (Figure 4), and, firstly, the apoptosis rate was detected. FACS analysis showed that the ESC apoptosis rate was significantly increased (*p* < 0.05) after transfection with siRNA-ESR1 (Figure 4a). Secondly, the cell cycles of sheep ESCs transfected with siRNA-ESR1 were compared. When transfected with siRNA-ESR1, the percentage of cells was significantly decreased (*p* < 0.05) in the G0/G1 phase (Figure 4b). The relative expression levels of apoptosis- and cell cycle- related genes were evaluated. As shown in Figure 4c,d, the relative expression of the genes Caspase3 (*p* < 0.01), Caspase9 (*p* < 0.01), P21 (*p* < 0.05), and P53 (*p* < 0.01) increased once the ESR1 gene had been interfered with (Figure 4c). The relative expressions of several genes related to the cell cycle, including Cyclin D1, Cyclin D2, Cyclin E1, CDK4, and CDK6, were tested. Transfection with siRNA-ESR1 significantly reduced the expression of Cyclin D1, Cyclin D2, Cyclin E1, and CDK6 (*p* < 0.01) (Figure 4d). Thus, ESR1 suppresses the apoptosis of Hu sheep ESCs in vitro. Transfection with siRNA-ESR1 inhibited cell cycle progression, indicating that ESR1 promotes cell cycle progression.

### 2.5. ESR1 Knockdown Inhibits Migration of Hu Sheep ESCs In Vitro

**Figure 5:**
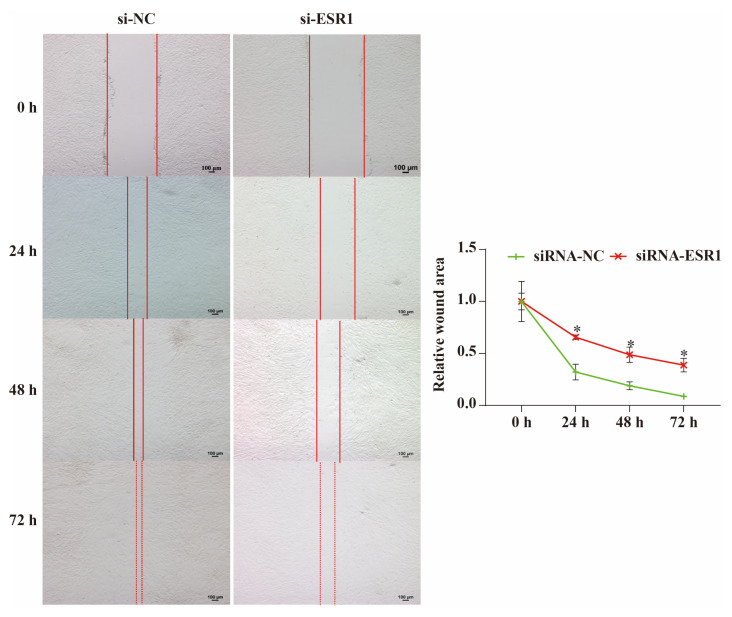
Analysis of roles of ESR1 in the migration of endometrial stromal cells (ESCs) using a wound-healing assay. Bar = 100 μm. * *p* < 0.05.

The wound assay results showed that the relative scratch width gradually decreased as the incubation time increased (Figure 5). If ESR1 was knocked down in ESCs, the relative scratch widths at 24, 48, and 72 h were significantly larger (*p* < 0.05) than those of the non-knocked-down cells. Therefore, we can conclude that ESC migration is inhibited when ESR1 is disrupted and that ESR1 can promote ESC migration in vitro.

## 3. Discussion

ESR1 is expressed in all cell layers in the uterus, such as the epithelium, stroma, and myometrium, and it is the main subtype [9]. ESR1 knockout in the endometrial epithelium, stroma, cavity, and glandular epithelium leads to abnormal decidualization in mice [10]. Although domestic ruminants do not undergo decidualization during implantation, the structure of the endometrium has common features across all species, and the initial events of blastocyst implantation are common among species [11]. Therefore, we hypothesised that ESR1 would play a crucial role in uterine function. In this study, we detected the relative expression levels of ESR1 in different organs in Hu sheep and observed that ESR1 was highly expressed in reproductive organs such as the endometrium, uterus, cervix, oviduct, and ovary. The relative expression levels of ESR1 and of ESR1-encoded ERα in the endometrium in high-reproductive Hu sheep were higher than those in low-reproductive Hu sheep, suggesting that ESR1 may play an important role in sheep reproductive performance, especially in the uterus. Based on Figure 1b, ESCs were selected to investigate the function of ESR1 in the sheep uterus, and ESR1 gene function was investigated by comparing the differences between ESCs with and without ESR1 knockdown.

Many researchers have reported that ERα can promote cell proliferation. In mouse endometriosis, oestrogen leads to a significant increase in cell height and proliferation through its receptor ERα, indicating that ERα promotes endometrial proliferation [12]. In uterine leiomyomas (ULMs), the upregulation of ERα expression promotes ULM cell proliferation [13]. Similar results were observed in our research. PCNA is used as a biomarker for cell proliferation [14]. In our study, the knockdown of ESR1 resulted in a decrease in PCNA expression in ESCs at both the gene and protein levels, indicating that ESC proliferation was inhibited, which is consistent with the results regarding EdU detection.

Caspase3/7 is activated in response to extracellular cell death-inducing cytokines [15]. In addition, apoptosis dependent on Caspase9 is initiated by DNA damage, endoplasmic reticulum stress, reactive oxygen species accumulation, and growth factor deficiency [16]. PCNA plays an important role in regulating DNA repair and replication [17]. In this study, after interfering with the expression of ESR1, the expression of Capase3 and Caspase9 in ESCs was upregulated, while the expression of PCNA was downregulated; hence, the cells may have undergone DNA damage, leading to an increase in cell apoptosis, as detected using flow cytometry. A critical step in the Caspase9-mediated apoptotic pathway is the regulation of Bcl-2 family proteins [18]. Bcl-2 and Bax belong to the Bcl-2 family [19], and their interaction maintains the integrity of the outer mitochondrial membrane [20] to inhibit the release of apoptotic proteins that activate caspases [21]. No remarkable changes were observed in the expression of Bcl-2 and Bax, indicating that the mitochondria might not have been affected by the treatment.

The eukaryotic cell cycle is mainly regulated by the cyclin-dependent kinase (CDK)–cyclin complex [22]. In mammalian cells, the CDK4/CDK6–Cyclin D complex activates and drives G1-phase progression and the CDK2–Cyclin E complex regulates the transition from the G1 to the S phase, while the CDK2–Cyclin A complex controls the S and G2 phases [23]. P53 is a transcription factor, and P21, a CDK1/2/4/6 inhibitor that halts cell cycle progression, is one of the main transcriptional targets of P53 [24]. The P53/P21 pathway can inhibit cell cycle progression by blocking CDK activity [25]. In the current study, the cell cycle was arrested in the G0/G1 phase after interference with ESR1, possibly because of the upregulation of P53 and P21 expression, which inhibited CDK4/6 activity. This result is similar to the findings of JavanMoghadam S. et al., who stated that in ESR1-knockdown MCF-7 cells, the cell cycle is arrested in the G2/M phase [26]. In addition, ERα has been shown to increase the expression of Cyclin D and CDK4/6, promoting the transition of MCF-7 cells from the G1 to the S phase of the cell cycle [27]. Similarly, Cyclin D1/2 was downregulated after ESR1 was knocked down in our study. Therefore, ESR1 is believed to promote the cell cycle by regulating Cyclin D and CDKs.

Normally, cells migrate collectively to form tissues and organs and to heal wounds [28]. Studies have shown that a thin endometrium is an important risk factor in implantation failure [29]. When the endometrial thickness is less than 7 mm, a positive correlation has been reported between thickness and implantation failure [30]. In mice, enhanced ESC activity and migration promote the recovery of endometrial function and increase fertility, possibly due to enhanced mesenchymal–epithelial transition in ESCs [29]. In our study, after the expression of ESR1 in ESCs was reduced, the healing speed of scratches decreased. Likewise, the inhibition of ESR1 expression led to suppressed migration and invasion in breast cancer cells [31]. Thus, ESR1 can promote the migration of ESCs, which is beneficial in maintaining the normal function of the endometrium.

However, it should be pointed out that we only studied the phenotypic effects of ESR1 gene knockdown on ESCs and did not investigate the overexpression of the ESR1 gene, meaning that our results are not entirely certain. Further research should be conducted in vivo, and the ESR1 gene should be knocked down and overexpressed to clarify the mechanism by which this gene affects sheep fertility.

## 4. Materials and Methods

### 4.1. Tissue Sample Collection

According to three successive lambing records and FecB genotype [32], nine healthy multiparous ewes (approximately 55 kg) were divided into three groups, HBB, LBB, and LB+, with three ewes in each group. HBB indicates high prolificacy and homozygosity with the FecB mutation (litter size = 3). LBB or LB+ indicates low prolificacy with homozygous or heterozygous FecB mutations (litter size = 1). To ensure that the ewes were in a similar physiological state when the samples were collected, sampling was conducted during the subsequent oestrus period after synchronous oestrus. The ewes were treated with vaginal sponge suppositories containing progesterone for 11 days. Each ewe was injected with 0.2 mg cloprostenol at the time of sponge removal. A ram was used to determine whether the ewes were in oestrus, and the time spent in oestrus was recorded. The ewes were slaughtered during the next oestrus to collect samples. After slaughter, endometrial samples were scraped from one of the uterine horns on the same side and position for each sheep and immediately stored in liquid nitrogen for subsequent analysis. The other horn was secured with 4% paraformaldehyde for immunohistochemical analysis [33]. Simultaneously, approximately 1 cm^3^ of samples was collected from other visceral tissues, including the liver, heart, spleen, kidney, cervix, uterus, oviduct, and ovary, and stored in liquid nitrogen.

### 4.2. Cell Isolation and Culture

A uterine horn was collected from a non-pregnant Hu sheep with high fecundity and transported to the laboratory under aseptic conditions. Sheep ESCs were obtained and identified as previously reported [34,35,36]. DMEM/F12 medium (Invitrogen, Carlsbad, CA, USA) with a foetal bovine serum volume fraction of 15% was used to culture the cells. The culture conditions were as follows: the temperature was 37 °C, and the CO_2_ density was 5%.

### 4.3. Total RNA of Tissues, Cell Isolation, and qRT-PCR

Total RNA was extracted from the samples using TRIzol reagent (Invitrogen, Waltham, CA, USA) according to the manufacturer’s instructions and evaluated for concentration and purity [37]. Subsequently, qualified total RNA samples were reverse transcribed into cDNA using reverse transcriptase (Vazyme, Nanjing, China) and were frozen at −20 °C for future use.

Primers were designed and assessed using the NCBI database. Primer pairs were used for quantification when the PCR amplification efficiency was between 0.9 and 1.1. The primer pairs used in this study are listed in Table 1. SYBR Green (Vazyme, Nanjing, China) was used for RT-qPCR in a reaction volume of 20 μL [38].

The relative gene expression levels of SUZ12 (endometrial tissue samples) or β-actin (other samples) were quantified using the 2^−ΔΔCT^ method. The PCR amplifications were carried out in three stages: the 1st stage was the hold stage, with the temperature maintained at 95 °C for 5 min; the 2nd stage was the PCR stage, with a programme cycle of 40 cycles at 95 °C for 10 s, 60 °C for 30 s, and 95 °C for 15 s; and the 3rd stage was the melt curve stage, with the temperature maintained at 95 °C for 15 s.

### 4.4. Immunohistochemistry

Uterine horn samples were fixed in 4% paraformaldehyde, and paraffin sections were then prepared. The samples were baked at 60 °C for 30 min for fixing and then de-paraffinised with xylene and hydrated using a gradient ethanol solution. Next, the slices were boiled in citrate buffer solution for 10 min and allowed to cool naturally for antigen retrieval. The cells were then treated with 3% H_2_O_2_ for 10 min and blocked with 5% BSA for 20 min to block endogenous peroxidase and reduce non-specific binding. The sections were incubated overnight at 4 °C with anti-ERα primary monoclonal mouse antibodies (1:250 dilution, Santa Cruz, Dallas, TX, USA). After incubation, the sections were rinsed three times with 1× PBS and then incubated with a secondary antibody (diluted 200 times, Biosynthesis Biotechnology, Beijing, China), homologous to the primary antibody, at 37 °C. PBS was used as the primary antibody in negative controls. Finally, paraffin sections were stained with 3,3′-diaminobenzidine (Beyotime, Haimen, China) at room temperature, and images were captured using a microscope (Nikon, Tokyo, Japan).

### 4.5. Western Blotting

Firstly, tissue or cell samples were lysed with an appropriate amount of RIPA protein lysis buffer (Beyotime, Shanghai, China), and 1 mM phenylmethylsulphonyl fluoride was added a few minutes before use. This step was performed on ice for 30 min, and the tissue samples were homogenised before lysis. Secondly, the samples were centrifuged, and the supernatant was collected for subsequent experiments. The protein concentration in the supernatant was determined using the BCA method (BCA assay kit; Beyotime, Shanghai, China). Thirdly, proteins were separated based on their molecular size using a 12% polyacrylamide gel containing sodium dodecyl sulphate. After electrophoresis, proteins were transferred to PVDF membranes (Millipore, Billerica, MA, USA), and the membranes were sealed with 5% (*w*/*v*) skim milk powder prepared with TBST for 2 h for blocking the non-specific binding sites. The membranes were then incubated sequentially with primary and secondary antibodies. The PVDF membranes were imaged using an imaging system (Fujifilm, Tokyo, Japan), and the relative expression levels of the target proteins were analysed using Image J software (version: 1.42; National Institutes of Health, Bethesda, MD, USA) based on the grayscale values in the image. The details of the antibodies used for immunohistochemistry and immunofluorescence are listed in Table 2.

### 4.6. siRNA Transfection in ESCs

The cells were cultured in 6-well plates after resuscitation. Small interfering RNAs (siRNAs) were synthesised according to the sequences shown in Table 3. When the degree of growth convergence reached 70–90%, the cells were transfected with siRNAs-ESR1 using the Lipofectamine 3000 reagent (Invitrogen, Carlsbad, CA, USA) according to the manufacturer’s instructions. Cells were collected 24 h after transfection for further analysis.

### 4.7. Analysis of Cell Proliferation

Proliferation capacities were assessed in both ESR1-knockdown and control ESCs to evaluate gene function by detecting DNA synthesis (EdU assay) (KeyGen, Nanjing, China) according to the manufacturer’s instructions.

### 4.8. Analysis of Apoptosis and Cell Cycle

Changes in apoptosis and cell cycle were detected using flow cytometry after interfering with the ESR1 gene in ESCs. Briefly, cells transfected with siRNA-ESR1 were collected and rinsed three times with DPBS. To detect apoptosis, the cells were resuspended in pre-cooled 1× binding buffer, and the concentration was adjusted to 1 × 10^6^ cells/mL. A 100 μL cell suspension (approximately 1 × 10^5^ cells) was transferred to a flow tube, mixed with 5 μL of Annexin V-FITC reagent, and incubated at room temperature for 15 min. Next, 5 μL of propidium iodide reagent (PI) was added, gentle mixing was performed, and the samples were incubated on ice for 5 min. All experiments were conducted in the dark. Finally, 400 μL of binding buffer was added, gentle mixing was performed, and the samples were immediately analysed to assess apoptosis (BD Biosciences, Franklin Lake, NJ, USA). For cell cycle analysis, harvested cells were first resuspended in DPBS, followed by the dropwise addition of ice-cold 70% ethanol (−20 °C) with continuous vortexing. After overnight fixation at 4 °C, the samples were centrifuged to remove ethanol and washed 1–2 times with DPBS. Subsequently, 500 μL of RNase A (final concentration, 50–100 μg/mL) was added, followed by incubation in a 37 °C water bath for 30 min. Finally, PI staining solution (final concentration 50 μg/mL) was added to the system, and the cells were incubated at room temperature for 15–30 min before flow cytometry analysis to evaluate the cells and identify their stages. To ensure reliability, cell cycle detection requires at least 10,000 cells in each sample.

### 4.9. Wound Assay

After thawing, ESCs were seeded in 6-well plates at a density of 1 × 10^6^ cells/mL and cultured in a 37 °C, 5% CO_2_ incubator. Upon reaching complete monolayer adherence, straight lines were scratched vertically to the bottom of the plate using a sterile 1 mL pipette tip. The wells were then gently washed 2–3 times with DPBS to remove detached cells and debris. Finally, the medium was replaced with a serum-free medium to minimise the effects of proliferation on cell migration. After scratching, images were captured every 24 h to document the wound’s width. The relative scratch width was defined as the ratio of the scratch width at different time points to the initial scratch width. Wound width was quantified using Image-Pro Plus image processing and analysis tools (Media Cybernetics, Rockville, MD, USA).

### 4.10. Statistical Analysis

Statistical analyses were performed using SPSS software (version 17.0; SPSS Inc., Chicago, IL, USA). Each experiment was conducted a minimum of three times, and the data were expressed as the mean ± SD. Student’s *t*-tests were used for comparisons between two independent groups. Statistical significance was set at *p* < 0.05.

## 5. Conclusions

In conclusion, this study demonstrates that ESR1 is a critical regulator of ovine endometrial development, orchestrating key cellular processes including proliferation, apoptosis, cell cycle progression, and migration. More significantly, our findings establish a compelling link between elevated endometrial ESR1 expression and superior reproductive performance in sheep. This novel association positions ESR1 as not merely a functional mediator within the endometrium but also a potential biomarker and pivotal factor in the genetic basis of high fecundity in sheep. Consequently, this work transcends mechanistic details to underscore ESR1’s broader biological significance in shaping ovine reproductive efficiency. Future research should focus on validating ESR1’s utility as a selection marker and elucidating the precise molecular pathways through which it governs fertility differences.

## Figures and Tables

**Table 1:** Information about primer pair sequences.

Item	Primer Sequences (5′-3′)	Fragment Size (bp)	Gene Bank No.
SUZ12	F: CTTTGAGAAACCAACGCAGATCTAT	207	XM_015098517.1
	R: TGCAGATGAGCTGACAAGCTA		
β-actin	F: TCAGCAAGCAGGAGTACGAC R: ACGAGGCCAATCTCATCTCG	138	NM_001009784.3
ESR1	F: TCTGGAAGAGAAGGACCAC R: AAGTGAGAGAGGAGGAGGAG	138	XM_042253634.1
P53	F: TTCCCCTTCCCTCAACAAGC R: GCGCGTAAATTCCCTTCCAC	143	NM_001009403.1
Bax	F: CGAGTGGCGGCTGAAAT	286	XM_015100640.1
	R: GGTCTGCCATGTGGGTGTC		
Bcl-2	F: CGCATCGTGGCCTTCTTT	113	XM_012103831.2
	R: CGGTTCAGGTACTCGGTCATC		
Caspase3	F: GGCTCTGAGTGTTTGGGGAA	131	XM_015104560.1
	R: CCTGGACAAAGTTCCGTGGT		
Caspase9	F: GCCAAGCCAAGGAAAACTCG R: CACGGCAGAAGTTCACGTTG	236	XM_012187488.2
P21	F: TGCCGCTGCCTCTTTGGT R: AAAGTCGAAGTTCCATCGCTCT	108	XM_012100423.3
CDK4	F: GCTGCTGCTGGAGATGCTGAC R: CTCTGCGTCACCTTCTGCCTTG	100	XM_012158548.3
CDK6	F: TCATTCTCACCGAGTGGTGC R: ATAGCTGGACTGCAGGAGGA	181	XM_012177413.4
CyclinD1	F: ACATGGAGCTGGTCCTGGTGA R: GGAGGGTGGGTTGGAAATGAA	188	XM_015102997.1
CyclinD2	F: AGCACGCTCAGACCTTCATC R: AGGCAATCCACATCCGTGTT	193	NM_001127290.1
CyclinE1	F: TTGCTGCTTCCGCCTTGTATC R: ACCATCCACTTGACACACTTCTC	100	XM_060398020.1

**Table 2:** Details of antibodies.

Antibody	Cat No.	Source	Dilution of IHC/IF	Dilution of WB
ERα	sc-787	Sant Cruz, Dallas, TX, USA	1:250	1:1000
β-actin	T0022	Affinity Biosciences, Cincinnat, OH, USA	-	1:5000
PCNA	ab15497	Abcam, Cambridge, UK	-	1:500
Goat anti-rabbit IgG	SA00001-2	Proteintech, Chicago, IL, USA	-	1:5000
Goat anti-mouse IgG	SA00001-1	Proteintech, Chicago, IL, USA	-	1:5000

**Table 3:** Sequences of siRNA.

Item	Sequences (5′-3′)
siRNA-ESR1-1053	Sense: GGAGAAUGUUGAAGCACAATT
	Antisense: UUGUGCUUCAACAUUCUCCTT

## Data Availability

The article described entirely theoretical research.
